# Stages of change and self-efficacy are related to consumption of food markers among Brazilian adolescents and young adults

**DOI:** 10.3389/fpubh.2022.1006898

**Published:** 2023-01-05

**Authors:** Stefany Corrêa Lima, Giselle Rhaisa do Amaral e Melo, Raquel Machado Schincaglia, Aline Cristine Souza Lopes, Natacha Toral

**Affiliations:** ^1^Center for Epidemiological Health and Nutrition Studies-NESNUT, Graduate Program in Human Nutrition, University of Brasília, Brasília, Brazil; ^2^Nutrition Interventions Research Group-GIN, Graduate Program in Nutrition and Health, Federal University of Minas Gerais, Belo Horizonte, Minas Gerais, Brazil

**Keywords:** transtheoretical model (TTM), food consumption, nutritional knowledge, self-efficacy, diet, stages of change, eating behavior, adolescents

## Abstract

**Objective:**

This study aimed at analyzing the association between stages of change, consumption of food markers, and self-efficacy in the adoption of healthy eating practices, adjusted by nutritional knowledge, among Brazilian adolescents and young adults.

**Methods:**

A cross-sectional analysis was conducted with 347 individuals from schools in the Federal District, Brazil. They completed a self-administered questionnaire covering: consumption of food markers, stage of change, self-efficacy in the adoption of healthy eating practices, and nutritional knowledge. Adjusted logistic regression was conducted.

**Results:**

Participants in pre-contemplation (OR = 0.22), contemplation (OR = 0.19), decision (OR = 0.13) and action (OR = 0.40) stages have less chance to have healthy eating than those in maintenance, including fruits and vegetables [pre-contemplation (OR = 0.23), contemplation (OR = 0.19), and decision (OR = 0.09)]. Adolescents and young adults in pre-contemplation (OR = 0.29) and contemplation (OR = 0.37) had lower chances of having low consumption of sugar-sweetened beverages compared to those in maintenance (*p* < 0.05). Adolescents and young adults in pre-contemplation (OR = 0.38) and contemplation (OR = 0.36) were less likely to have high self-efficacy scores than those in maintenance (*p* < 0.05). Higher score of self-efficacy was associated with a lower chance of having a high consumption of sugar-sweetened beverages (OR = 1.02; *p* = 0.032).

**Conclusion:**

Regardless of nutritional knowledge, individuals in the earlier stages of change are less likely to have an adequate consumption of healthy foods markers, including fruits and vegetables, and low sugar-sweetened beverages consumption. They are also less likely to have high self-efficacy scores than those in maintenance. Nutritional interventions to focus on enhancing self-efficacy among adolescents and young adults in earlier stages of change to improve dietary habits.

## 1. Introduction

The prevalence of overweight and obesity is increasing at an alarming rate in adolescents and young adults worldwide (1). One of the main factors that underlie this situation is that suggested dietary guidelines are rarely met by them (2–5). This non-adherence is associated with many negative health effects, that could be expressed even in early life (6, 7). In addition, poor dietary habits during adolescence may be carried into adulthood, leading to other chronic diseases, reducing the quality of life (6, 8). Thus, it is crucial to promote healthy eating to prevent diseases and ensure food and nutritional security at this stage of development (7, 9).

However, the promotion of healthy dietary habits requires an understanding of the variables that impact adolescents and young adults' eating behavior at different levels. The transtheoretical model (TTM) stands out as a way to understand and guide strategies to modify eating behaviors. The theory proposes that behavioral change occurs over time, and it is mediated by different constructs. The stages of change are one of them and they are the central component of the model (10). Each stage represents different perceptions and degrees of motivation to modify one's diet. They include *precontemplation* (no intention to improve diet in the near future), *contemplation* (beginning to consider diet changes, but many barriers are identified), *decision* (preparation and confidence to improve diet), *action* (visible and concrete, although recent, diet changes), and *maintenance* (diet changes sustained for a more significant time, usually more than 6 months) (11). Another construct of the TTM is self-efficacy, that is the individual's confidence to handle situations without relapsing to former behavior. When moving forward through the stages, self-efficacy is expected to increase (10).

A cross-sectional study with adolescents in Hangzhou, China, found that progression through stages and higher scores on self-efficacy predicted higher consumption of fruits and vegetables (12). Similar results were also found in other studies with adolescents (13, 14). Nutritional knowledge is also a determinant that could influence changes in dietary habits (15). Individuals with more nutritional knowledge are more likely to consume sufficient amounts of fruits, vegetables and less fat, compared to those with a lower level of knowledge (16). Increasing knowledge may eventually improve the diet of target populations (17).

To our knowledge, no previous studies have examined, simultaneously, the association of food consumption, stages of change, self-efficacy for healthy eating and nutritional knowledge among adolescents and young adults. The use of the nutritional knowledge as an adjustment variable is important to identify the real association between diet and TTM constructs. This adjustment was performed in a previous study (18). Considering that nutritional knowledge is important to adopt healthy eating habits, but not enough, it is important to conduct analyzes with the adjustment by nutritional knowledge to remove possible effects of this variable and evaluate how the TTM constructs are implied in healthy eating. Our hypothesis is that moving forward through the stages of change and increasing scores of self-efficacy are associated with better consumption of food, regardless of nutritional knowledge.

Therefore, this study aimed at analyzing the association between stages of change, food consumption, and self-efficacy, adjusted by nutritional knowledge, in the adoption of healthy eating practices among Brazilian adolescents and young adults.

## 2. Materials and methods

### 2.1. Study design, data collection, and participants

This is a cross-sectional study with baseline data from a nutritional intervention with Brazilian adolescents and young adults conduced in 2019 and registered in the Brazilian Clinical Trials Registry (No. RBR-5b9jk7, trial: http://www.ensaiosclinicos.gov.br/rg/RBR-5b9jk7/). The protocol of this intervention has been reported elsewhere (11). The current analysis examined consumption of food markers, stages of change, self-efficacy for adopting healthy eating practices and nutritional knowledge among individuals aged 16–21 years old. This study was conducted according to the guidelines laid down in the Declaration of Helsinki and was approved by the University of Brasilia's Research Ethics Committee (No. 2,839.510). Written informed consent was obtained from school principals, adolescent's parents and young adults. Adolescents gave their assent too. The reporting of this study follows the STROBE-nut framework (19).

Senior students were recruited from the last year of seven public high schools of the Federal District, Brazil, that participated in the Health in School Program (HSP, in Portuguese Programa Saúde na Escola) (20). The inclusion criteria were: possession of a smartphone and use of WhatsApp (11), which was necessary for the nutritional intervention, but not explored in this article.

### 2.2. Sample size and recruitment

The population considered was the total number of senior students from the 19 schools in the Federal District that joined the HSP in 2018-2019, according to information provided by the State Health Secretariat of the Federal District, but excluding rural and night schools. The sample calculation was performed considering the population of 4,183 participants, the distribution of Brazilian adolescents and young adults in the stages of change observed in the literature, in which 68% were in pre-contemplation and contemplation stages (21), a sampling error of 5% and a 95% confidence level. Thus, the minimum sample size calculated was 310 participants, to which 10% were added to cover possible losses and inconsistencies of questionnaires, totaling 341 participants.

School recruitment and data collection were conducted from June to August 2019 by previously trained researchers. A statistician used a computer-generated randomization method to select schools to participate in the study. Principals were visited in the first phase of the recruitment process. Second phase included explanation of the study for students and delivery of consent and assent terms to be signed within 7 days. New schools were drawn until sample was reached. Data collection took place inside the classrooms, during school hours, in a single day (11).

### 2.3. Measures

A self-administered questionnaire was applied at schools. Variables investigated were: age, gender, consumption of food markers (healthy vs. unhealthy), stage of change, self-efficacy in the adoption of healthy eating practices, and nutritional knowledge. Maternal education was assessed through a multiple-choice question used in national population surveys (22).

To assess consumption of food markers, a questionnaire was applied, based on an annual national survey, including 13 and 10 markers of healthy and unhealthy eating, respectively. Participants had to mark if those particular food groups of items were eaten in the day before (23).

To evaluate the stages of change, an algorithm described by López-Azpiazu et al. in their study with Spanish adolescents was translated to Portuguese (24). The instrument classified adolescents in stages based on a general diet and in accordance with the participant's own perception. Respondents who reported they have never pursued healthy eating habits were asked if they have considered changing their diet in the last month: negative responses were classified as pre-contemplation; positive responses were classified as contemplation – if the participant did not have the confidence to make their diet healthy in the next month, or decision – if the participant had the confidence to make their diet healthy in the next month. Those who reported they were trying to have a healthier diet recently (<6 months) were classified in action. If they reported they were currently trying and/or have tried to eat a healthy diet for at least 6 months, they were considered in maintenance.

To assess self-efficacy and nutritional knowledge, an instrument developed and validated for a nutritional intervention with Brazilian adolescents by Chagas et al. (25) was used. For the self-efficacy module, the adolescents and young adults had to answer each of 19 questions about how much they intend to take different actions to adopt healthy behaviors, with a Likert scale ranging from 1 (“Definitely no”) to 5 (“Definitely yes”). For the nutritional knowledge module, the adolescents and young adults had to evaluate each of 15 statements about healthy eating on a Likert scale ranging from 1 (“I don't agree at all”) to 5 (“I totally agree”). The final version of the self-administered questionnaire can be found elsewhere (11).

### 2.4. Data analysis

KoBoToolbox software was used to complete the survey questionnaires (26). There were limited response options to minimize typing errors. For each item of consumption of food markers, the participant received one point if there was a consumption of that food item in the previous day, and from that, final scores for healthy and unhealthy eating were calculated by summing the items, so, the maximums were 13 and 10 points, respectively. In addition, other scores were calculated by grouping items like fruits and vegetables (maximum score of 6 points) and sugar-sweetened beverages–soda, industrialized juices and dairy sugary drinks (maximum score of 3 points). For the self-efficacy and nutritional knowledge scales, a final score was calculated with a maximum score of 95 and 75 points, respectively.

Descriptive and exploratory analyses were performed on each variable. We proceeded with an adjusted logistic regression analysis with an estimation of the Odds ratio (OR), confidence interval of 95%, and determination coefficient (R^2^). The outcomes were: (1) healthy eating, (2) unhealthy eating, (3) fruits and vegetables ingestion, (4) sugar-sweetened beverages ingestion, and (5) self-efficacy. Outcomes 1, 3, and 5 were considered adequate when adolescents and young adults were in the highest quartile, while outcomes 2 and 4 were considered adequate when adolescents and young adults were in the lowest quartile of consumption, therefore representing healthier eating. Independent variables, tested in separated models, were the classification of stages of change (categories - using maintenance as a comparison category) and self-efficacy (scores). Self-efficacy was used as an outcome and independent variable. Nutritional knowledge was selected as an adjustment variable since higher scores in that variable represent more probability to have better nutritional behaviors and consumption (18). Statistical analysis was performed using SPSS version 23.0, with a significance level of 5%.

## 3. Results

**Figure 1:**
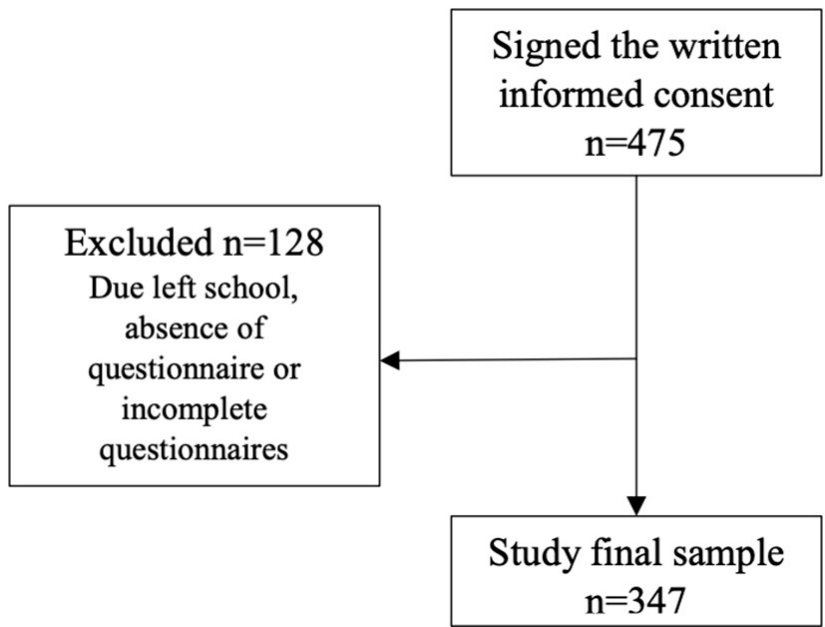
Flow diagram of participant inclusion process. Federal District, Brazil, 2019.

This study included 347 participants and Figure 1 shows the flowchart of the participant's inclusion process. The mean age was 18.04 (SD 0.75, min: 16; max: 21) years old and girls were 69.7% of the sample. Most participants were in the early stages of change (39.2 in contemplation and 22.5% in pre-contemplation), followed by action (17.9%), maintenance (10.3%) and preparation (10.1%).

The highest quartiles of the outcomes included participants with an intake of 8 or more healthy food items; 3 or more fruits and vegetables; and 78 or more points for self-efficacy. The lowest quartiles included participants with 2 or fewer unhealthy food items; and 0 sugar-sweetened beverages (Table 1).

**Table 1:** Distribution of the participants' consumption of food markers, food groups consumption, and self-efficacy in the adoption of healthy eating practices according to the stages of change.

	**Pre-contemplation 78 (22.5)**	**Contemplation 136 (39.2)**	**Preparation 35 (10.1)**	**Action 62 (17.9)**	**Maintenance 36 (10.3)**	***p-value***
**Consumption of food markers**						
Healthy Eating (highest quartile: ≥8 food items)	11 (18,3)	17 (28,3)	3 (5,0)	14 (23,3)	15 (25,0)	0,001^1^
Unhealthy Eating (lowest quartile: ≤2 food items)	14 (26,9)	17 (32,7)	3 (5,8)	11 (21,2)	7 (13,5)	0,505^1^
**Food groups**							
Fruits and vegetables (highest quartile: ≥3 food items)	15 (19,0)	22 (27,8)	3 (3,8)	21 (26,6)	18 (22,8)	<0,001^1^
Sugar-sweetened beverages (lowest quartile:0 food item)	16 (16,3)	35 (35,7)	11 (11,2)	18 (18,4)	18 (18,4)	0,023^2^
Self-efficacy (highest quartile: ≥78 points)	13 (16,0)	23 (28,4)	15 (18,5)	16 (19,8)	14 (17,3)	0,002^2^

Federal District, Brazil, 2019. ^1^ - Fisher exact test; ^2^ - Pearson chi-squared test, both with 5% of significance.

Mean scores of healthy and unhealthy eating were 6.18 (SD 2.37) and 3.61 (SD 2.11), respectively. The mean scores of fruits and vegetables and sugar-sweetened beverages were 2.19 (SD 1.59) and 1.14 (SD 0.92), respectively. Mean scores of self-efficacy and nutritional knowledge were 67.86 (SD 12.92) and 65.96 (SD 8.25), respectively.

The analysis of logistic regression, adjusted by nutritional knowledge, is shown in Table 2. There was a statistically and significant association between the highest quartile of healthy eating and precontemplation stage (OR = 0.22; *p* = 0.002), contemplation stage (OR = 0.19; *p* < 0.001), decision stage (OR = 0.13; *p* = 0.003), and action stage (OR = 0,40; *p* = 0,047). Therefore, adolescents and young adults in pre-contemplation have a 78% lower chance of being in the highest quartile of healthy eating consumption when compared to those in the maintenance stage. The percentages reach 81, 87, and 60% lower chance of being in the highest quartile of healthy eating regarding adolescents and young adults in contemplation, decision, and action respectively, when compared to those in the maintenance stage (Table 2).

**Table 2:** Association between consumption of food markers and self-efficacy (outcomes) with stages of change (independent variable) among Brazilian adolescents and young adults.

	Odds Ratio**	**Confidence interval** **95%**		**R^2^ (%)**	***p*-value**
		**Minimum**	**Maximum**		
**1. Healthy eating**					
Stages of change (Reference: maintenance)*				8.5	
Pre-contemplation	0.22	0.08	0.56		**0.002**
Contemplation	0.19	0.08	0.45		**<0.001**
Decision	0.13	0.03	0.50		**0.003**
Action	0.40	0.16	0.98		**0.047**
**2. Unhealthy eating**					
Stages of change (Reference: maintenance)*				2.8	
Pre-contemplation	1.01	0.36	2.79		0.991
Contemplation	0.63	0.23	1.67		0.356
Decision	0.39	0.09	1.67		0.207
Action	0.93	0.32	2.67		0.896
**3. Sugar-sweetenedbeverages**					
Stages of change (Reference: maintenance)*				7.4	
Pre-contemplation	0.29	0.12	0.70		**0.006**
Contemplation	0.37	0.17	0.81		**0.013**
Decision	0.46	0.17	1.23		0.125
Action	0.42	0.18	1.10		0.054
**4. Fruits and vegetables**					
Stages of change (Reference: maintenance)*				11.0	
Pre-contemplation	0.23	0.09	0.55		**0.001**
Contemplation	0.19	0.08	0.42		**<0.001**
Decision	0.09	0.02	0.36		**0.001**
Action	0.52	0.22	1.20		0.128
**5. Self-efficacy**					
Stages of change (Reference: maintenance)*				14.4	
Pre-contemplation	0.38	0.15	0.98		**0.046**
Contemplation	0.36	0.15	0.82		**0.016**
Decision	1.26	0.47	3.37		0.637
Action	0.59	0.23	1.45		0.254

Federal District, Brazil, 2019. Adjusted logistic regression models with an estimation of the ^**^ Odds ratio (OR), confidence interval of 95%, and determination coefficient (R^2^ in %). The outcomes 1, 3, and 5 were considered adequate when adolescents and young adults were in the highest quartile, while outcomes 2 and 4 were considered adequate when adolescents and young adults were in the lowest quartile of consumption, therefore representing healthier habits. ^*^Adjusted models by nutritional knowledge. Numbers in bold are statistically significant.

No association was found between the total score of unhealthy eating and stages of change. However, adolescents and young adults in pre-contemplation or contemplation stages have a 71% (OR = 0.29; *p* = 0.006) and 63% (OR = 0.37; *p* = 0.013) lower chance, respectively, of being in the lowest quartile of sugar-sweetened beverages consumption when compared to those in the maintenance stage (Table 2).

Adolescents and young adults in pre-contemplation have a 77% lower chance of being in the highest quartile of fruits and vegetables consumption when compared to those in the maintenance stage (OR = 0.23; *p* = 0.001). The corresponding value for adolescents and young adults in contemplation was 81% (OR = 0.19; *p* < 0.001) and 91% (OR = 0.09, *p* = 0.001) for adolescents and young adults in preparation both compared to maintenance stage (Table 2).

Regarding the association between self-efficacy and stages of change, it was found that adolescents and young adults in pre-contemplation and contemplation have a 62% (OR = 0.38; *p* = 0.046) and 64% (OR = 0.36; *p* = 0.016) lower chance, respectively, of being in the highest quartile of self-efficacy when compared to those in the maintenance stage (Table 2).

The only significant association found between self-efficacy and consumption of food markers was related to the sugar-sweetened beverages ingestion. For each point of increase in the self-efficacy score, there was a 2% increase in the chance of the adolescent belonging to the first quartile of sugar-sweetened beverage ingestion (OR = 1.02; *p* = 0.032; Table 3).

**Table 3:** Association between the consumption of food markers (outcome) and self-efficacy (independent variable) among Brazilian adolescents and young adults.

	Odds Ratio**	**Confidence interval 95%**		**R^2^ (%)**	***p*-value**
		**Minimum**	**Maximum**		
1. Healthy Eating*	1.00	0.98	1.02	0.1	0.707
2. Unhealthy Eating*	1.01	0.98	1,03	1.5	0.364
3. Sugar-sweetened beverages*	1.02	1.00	1.04	6.0	**0.032**
4. Fruits and vegetables*	1.00	0.98	1.03	0.3	0.397

Federal District, Brazil, 2019. Adjusted logistic regression models with an estimation of the ^**^ Odds ratio (OR), confidence interval of 95%, and determination coefficient (R^2^ in %). The outcomes 1, 3 and 5 were considered adequate when adolescents and young adults were in the highest quartile, while outcomes 2 and 4 were considered adequate when adolescents and young adults were in the lowest quartile of consumption, therefore representing healthier habits. ^*^Adjusted models by nutritional knowledge. Numbers in bold are statistically significant.

## 4. Discussion

Our study showed that most of the participants were classified in the earlier stages of change (61.7% in pre-contemplation and contemplation stages), as expected, considering that Cunha et al. (21) found 68% of Brazilian adolescents and in pre-contemplation and contemplation stages. Participants' self-efficacy in the adoption of healthy eating practices and nutritional knowledge were very high. This is also similar to others studies with adolescents regarding mean scores' results of self-efficacy (25) and nutritional knowledge (25, 27, 28).

This research found that, regardless the nutritional knowledge, adolescents and young adults in the first stages of change are less likely to have an adequate consumption of healthy foods markers, including fruits and vegetables, than those in maintenance. It was also found that the higher the self-efficacy score, the lower the chance of having a high consumption of sugar-sweetened beverages. Finally, adolescents and young adults in the pre-contemplation and contemplation stages were significantly less likely to have high self-efficacy scores than those in maintenance. These findings are in consonance with the TTM because, as the individual moves forward through the stages of change, self-efficacy is expected to improve compared to earlier stages. Moreover, according to the model, low consumption of healthy food is expected to be found at earlier stages and previous studies with adolescents and young adults have shown that subjects in the action and maintenance stage are likely to have higher fruits and vegetables intake than subjects in other stages (13, 29). Davoodi et al. (13) performed a research with 345 students studying in eight high schools in Bandar Abbas, Iran, and indicated that the individuals' progress along the stages of change from pre-contemplation to maintenance level was associated with a significant increase in fruit and vegetable consumption. However, none of them included the simultaneous evaluation of nutritional knowledge, as we did.

On the other hand, the first stages (pre-contemplation and contemplation) usually have a higher consumption of unhealthy food (30, 31). In our study, except for sugar-sweetened beverages, no difference was found for unhealthy eating regarding stages of change, controlling for nutritional knowledge. Our findings may indicate that adolescents and young adults who are at the final stages of the TTM increase the consumption of markers of healthy eating, but do not necessarily reduce the consumption of ultra-processed foods. A Brazilian national survey showed that adolescents and young adults have a habitual consumption of ultra-processed food (32), probably not only because of the appreciation of this type of food, but also for looking for social identification and approval, commonly found in this stage of life (33). Other variables might be involved in the maintenance of unhealthy eating across the stages of change, e.g., food price. Several studies have shown a connection between the price of unhealthy food and its consumption, especially by adolescents, which involves a complex interplay of economic factors that should be better analyzed by future studies (34–36).

The association between stages of change and self-efficacy was also expected since the TTM points out that moving forward through the stages of change implies better self-efficacy to adopt behavior changes (10). It was also found in previous studies with adolescents (13, 37, 38). However, the novelty of our study was finding that this relation was maintained even when nutritional knowledge was selected as an adjustment variable. Regardless of what adolescents and young adults know about diet, self-efficacy and stages of change are associated with eating behavior. Self-efficacy seems to play an important role in the adoption of healthy eating practices and it has also been mentioned in several previous studies as being the key factor in encouraging people to have healthy dietary behavior (39–41).

In this sense, it is crucial to promote healthy eating habits among adolescents and young adults through educational interventions to ensure food and nutritional quality at this life stage, mainly for those in the earlier stages of change. A recent systematic review found that interventions which aimed at specific stages of change led to lower fat intake and higher fruit and vegetable consumption (42). To the best of our knowledge, the current study also stands out for investigating, for the first time in Brazil, consumption of food markers, stage of change, self-efficacy in the adoption of healthy eating practices, and nutritional knowledge simultaneously. In this way, this research can help in the design of nutritional interventions aimed at this age group.

The limitations of the present study come from the cross-sectional nature of this research, which does not allow causal factors to be identified. On the other hand, observational studies can open hypotheses that improve the direction of future investigations. Besides, consumption of food markers data were reported by adolescents and young adults themselves and were related to the food eaten the day before, which implies possible bias. However, the choice of instrument to measure consumption of food markers was made based on a questionnaire used in national surveys (23), so that future analyses, and comparisons could be performed.

## 5. Conclusion

Regardless of the nutritional knowledge, adolescents and young adults in the earlier stages of change (pre-contemplation and contemplation) are less likely to have an adequate consumption of healthy foods markers, including fruits and vegetables, and low sugar-sweetened beverages consumption than those in maintenance. They are also less likely to have high self-efficacy scores than those in maintenance. Findings from this study may guide nutritional interventions to focus on enhancing self-efficacy among adolescents and young adults in earlier stages of change to improve dietary habits. Future studies should explore personal and environmental factors that contribute to adolescents and young adults ' unhealthy eating in all stages of change.

## Data availability statement

The original contributions presented in the study are included in the article/supplementary material, further inquiries can be directed to the corresponding authors.

## Ethics statement

The studies involving human participants were reviewed and approved by Comissão Ética em Pesquisa- Universidade de Brasília. Written informed consent to participate in this study was provided by the participants' legal guardian/next of kin.

## Author contributions

SL, GM, RS, and NT: drafting the work and analysis of data. AS and NT: design and critical review. All authors contributed to the article and approved the submitted version.
